# Incidence Rate for Hantavirus Infections without Pulmonary Syndrome, Panama

**DOI:** 10.3201/eid1710.101717

**Published:** 2011-10

**Authors:** Blas Armien, Juan M. Pascale, Carlos Munoz, Sang-Joon Lee, Kook L. Choi, Mario Avila, Candida Broce, Anibal G. Armien, Fernando Gracia, Brian Hjelle, Frederick Koster

**Affiliations:** Gorgas Memorial Institute for Health Research, Panama City, Panama (B. Armien, J.M. Pascale);; Ministry of Health, Panama City (C. Munoz, M. Avila, C. Broce);; University of New Mexico Cancer Center, Albuquerque, New Mexico, USA (S.-J. Lee, K.L. Choi);; Inje University, Gimhae, South Korea (K.L. Choi);; University of Minnesota, St. Paul, Minnesota, USA (A.G. Armien);; Santo Tomas Hospital, Panama City (F. Gracia);; University of New Mexico, Albuquerque (B. Hjelle);; Lovelace Respiratory Research Institute, Albuquerque (F. Koster)

**Keywords:** hantavirus, Bunyaviridae, epidemiology, hantavirus pulmonary syndrome, viruses, Panama, dispatch

## Abstract

During 2001–2007, to determine incidence of all hantavirus infections, including those without pulmonary syndrome, in western Panama, we conducted 11 communitywide surveys. Among 1,129 persons, antibody prevalence was 16.5%–60.4%. Repeat surveys of 476 found that patients who seroconverted outnumbered patients with hantavirus pulmonary syndrome by 14 to 1.

In the Americas, hantavirus species that occur at low frequency are associated with the severe disease hantavirus pulmonary syndrome (HPS) (*1**,**2*), and species that occur at higher frequency are associated with milder disease (*3**–**5*). In Panama, HPS is caused by the Choclo virus, for which a common rodent, the fulvous pygmy rice rat (*6*), is host. Serum antibody prevalence against this virus is 3%–33% in neighborhoods where HPS cases have occurred (*7*) and 16%–45% according to selected communitywide surveys (*8*). Neutralization-inhibition assays of antibody-positive serum indicated past infections caused by Choclo virus (*9*). To obtain a more accurate incidence of hantavirus infections in Panama, we conducted repeat surveys to identify hantavirus seroconversions during 1- to 3-year intervals between surveys. Our goal was to compare incidence of seroconversion with that of concurrent HPS in the same communities.

## The Study

During 2001–2007, a total of 4 communities (3 in Los Santos Province and 1 in Veraguas Province) within hantavirus-endemic agroecosystems in western Panama were sampled 2–4 times at 1- to 3-year intervals (Table 1). Informed written consent was obtained from all adult participants and from parents or legal guardians of minors. Consent and assent forms were reviewed and approved by institutional ethics review boards at the University of New Mexico, the Gorgas Memorial Institute in Panama City, and the protocol review committee of the International Centers for Infectious Diseases Research program of the National Institute of Allergy and Infectious Diseases. Eligible participants were all adults and children >2 years of age who permanently resided in each community according to the 2000 national census. The reasons for noninclusion in the first and subsequent surveys were absence during the week of the survey and refusal to participate.

**Table 1:** Hantavirus seroprevalence, western Panama*

Community and year of survey	Community population	Persons tested, no. (% of community)	No. (%) IgG positive†	No. undergoing follow-up testing‡	Repeated tests only: no. (%) IgG positive†§
Agua Buena					
2003	175	105 (60)	47 (44.8)	**–**	–
2004	175	108 (62)	59 (54.6)	75	41 (54.7)
2006	160	102 (64)	61 (60.4)	69	42 (61.8)¶
2007	164	99 (60)	49 (49.5)	55	33 (60.0)#
Isla Cañas					
2001	276	223 (81)	74 (33.2)	–	–
2003	184	120 (65)	56 (46.7)	90	44 (48.9)**
2006	187	120 (64)	63 (52.5)	49	26 (53.1)#
San Jose					
2001	593	486 (82)	80 (16.5)	–	–
2003	454	327 (72)	84 (25.7)	270	70 (25.7)#
Borracherones					
2003	85	61 (72)	19 (31.1)	–	–
2006	93	87 (94)	23 (26.4)	41	11 (26.8)

*Ig, immunoglobulin; –, not applicable. †No. IgG positive (point prevalence % of seropositivity). ‡No. persons tested in this and previous survey. §Increase for all 4 communities combined not significant. ¶Increase in seroprevalence significant (p = 0.007) by Fisher exact test. #Increase for combining Los Santos localities and year, p = 0.0014. **Increase significant (p = 0.001).

A questionnaire administered to the head of household asked for a history of respiratory-related illnesses and hospitalizations within the past 3 years. Venous blood was collected from all family members for serologic testing. Results of the surveys were provided to each participating community through community meetings. Surveillance for HPS was conducted in the same communities as the serosurvey and nationally through reports to the Ministry of Health, and cases of HPS were confirmed by questionnaire. The diagnosis of HPS required finding immunoglobulin (Ig) M in acute-phase serum, detection of Choclo virus RNA in serum by reverse transcription PCR, typical respiratory signs and symptoms, and chest radiographic findings compatible with pulmonary edema.

Heparinized whole blood collected by arm venipuncture was separated by centrifugation; plasma was stored at −20°C until analysis. In binding assays, antibody to all known hantaviruses indigenous to the Americas cross-react with the N protein of Sin Nombre virus (*10*). A strip immunoblot assay for IgG containing recombinant N protein of the 3H226 genotype of Sin Nombre virus was used as described (*10*); the criterion for positivity was a dark band for Sin Nombre N protein at a serum dilution of 1:200. An enzyme immunoassay used recombinant nucleocapsid protein from Sin Nombre virus (*11*); the cutoff value was established at 3 SD above reactivity to a panel of known positive serum. All samples were tested by both assays; the concordance of the enzyme immunoassay and strip immunoblot assay in this study was 97%, and the criterion for seropositivity was a positive reaction in both assays. Loss of antibody in persons with previously positive serum was determined by 2 independent tests with both assays. IgM against hantavirus was not tested. In Panama, all HPS patients tested have had positive reverse transcription PCR results for Choclo genomic RNA in acute-phase blood samples (*9*), and antibody has been detected by both assays.

Data were transferred from field collection forms to a database (Epi Info version 6.04d, Centers for Disease Control and Prevention, Atlanta, GA, USA) for statistical analyses using Epi Info software. Changes in seroprevalence within each community were tested by Fisher exact test for each interval and by longitudinal analysis for all intervals and communities by a generalized estimating equation (*12*). Increases in seroprevalence according to community and year of survey were tested by using analysis of covariance.

The 11 surveys repeatedly sampled 60%–85% of the total population of each community, for a total of 1,838 samples from 1,129 persons. Overall antibody prevalence was 32.9%, varying from 16.5% to 60.4% in individual surveys (Table 1). In each of the 3 Los Santos communities (Agua Buena, Isla Cañas, San Jose), seroprevalence increased annually by ≈5% (Table 1); the overall seroprevalence increases for the combined Los Santos communities were significant (Fisher exact test, p = 0.0014). The changes in seroprevalence were community specific (analysis of covariance *F* = 5.24, p = 0.0043), but increases in seroprevalence in the 4 communities combined was not (general estimating equation).

Among the study population, seroconversion was documented for 70 persons, and HPS was diagnosed for 5 other persons in the same communities during the intervals studied (Table 2). In the cohort of 476 persons in all 3 Los Santos communities sampled in 2 back-to-back surveys (Table 2), the 70 seroconversions occurred in persons in all age cohorts and equally among persons of both sexes (data not shown). No person who seroconverted gave a history of HPS-like illness or hospitalization for an acute respiratory illness. A separate study of outpatients with febrile illnesses was conducted in 4 clinics in the hantavirus-endemic area. This study found 48 adults and children with the typical febrile prodrome, unremarkable chest radiographs, and either serum IgM specific for hantavirus nucleoprotein or Choclo virus genomic RNA in the acute-phase blood sample (B. Armien and J.M. Pascale, unpub. data). These findings of symptomatic hantavirus infections confirm previous observations derived from neutralization-inhibition antibody assays (*9*).

**Table 2:** Hantavirus seroconversions and HPS cases, western Panama*

Community and survey intervals	No. undergoing follow-up testing†	No. seroconverted/ no. seronegative‡	No. conversions/ 100 person-years	No. seroreverted/ no. seropositive§	No. HPS cases¶
Agua Buena					
2003–2004	75	8/42		0/33	1
2004–2006	69	6/29		4/40	0
2006–2007	55	5/23		4/32	0
Total		19	15.4	8	1
Isla Cañas					
2001–2003	90	18/64		0/26	0
2003–2006	49	2/24		1/25	0
Total		20	10.0	1	0
San Jose					
2001–2003	270	29/228		1/42	3
Total		29	6.4	1	3
Borracherones					
2003–2006	41	2/26		6/15	1
Total		2	2.6	6	1
Study totals		70	8.2	16	5

*HPS, hantavirus pulmonary syndrome. †No.persons repeat tested in this and previous survey. ‡ No. seroconverted among subset who were seronegative at the beginning of the interval. § No. seroreverted among subset who were seropositive at the beginning of the interval. ¶HPS cases verified by Ministry of Health during interval in community.

The incidence of 70 seroconversions in 857 person-years of observation (Table 2) was equivalent to 8 infections per 100 person-years. The ratio of infection detected by seroconversion to infection resulting in HPS was 14:1. The mean ages of persons who seroconverted (43 years) and those with HPS (43 years) were the same. Undercounting of HPS cases was not likely because HPS is a highly publicized illness throughout Panama, and diagnostic serologic testing is readily available through the Ministry of Health.

A total of 16 seroreversions, compared with 70 seroconversions, occurred among persons in most age cohorts, mean age 48 years. For HPS caused by Sin Nombre and Andes viruses, serum antibody typically persists for years (*13*). Serum antibodies after mild or asymptomatic infections may not persist for many years.

## Conclusions

Antibody prevalence surveys are useful for identifying populations and locations at risk, monitoring changes in incidence, and focusing limited public health resources. Determining whether the observed increases in seroprevalence will be sustained requires additional surveys, but this information will be useful as the new agroeconomy increasingly emphasizes the monoculture of products (rice and sugar cane) favorable to rodents (*14*). Nonetheless, the documentation of large numbers of mild or asymptomatic hantavirus infections not progressing to HPS has identified a larger effect of this zoonotic disease.
